# Food Conditions in Germany

**Published:** 1919-10-11

**Authors:** 


### FOOD CONDITIONS IN GERMANY.

### II. A Situation the World must Realise.

As pointed out in Article I., two-thirds of the inhabitants of Germany have been chronically starved for three years, and have been able to obtain only about 2,000 calories of food per diem instead of 3,000 and upwards according to the amount of work to be done, and this starvation is the more serious since not only were the total calories very low, but certain food constituents which are irreplaceable were especially deficient—viz., proteins and fats. Carbohydrates (*i.e.*, starchy foods) were as a rule more easily obtainable, and these to a certain extent can replace fat, but protein, which is absolutely necessary for muscle repair, cannot be replaced by anything. The amount of protein and fat required for the average man per diem is 100 grammes of each, and during the three years since 1916 the German rations were about 45 grammes protein and 20 grammes of fat. The minimum protein which man should have, according to the Royal Society is 70 grammes, and, as pointed out in the report, they might possibly have managed with 45 grammes had the protein been animal protein; but it was mostly vegetable protein, which is not more than half the value of animal protein. The deficiency of fat was greatly emphasised when the tremendous shortage of milk is considered, which of course has had a most disastrous effect, especially, as will be pointed out, on children. The result of this shortage of food may be viewed from the effect it has on the (1) mental and (2) physical condition of the people. As regards the mental condition, the chief defect noted among the lower and middle classes was general apathy, listlessness, and hopelessness—no spirit of resentment was found among the workmen, but simply dull depression and apathy.

### Mental and Moral Prostration.

It is, however, " among the leading men that the mental and moral prostration is the most striking. They seem hopeless and despairing of any future for themselves or their country. This hopelessness is more striking than any resentment." The contrast of the mentality of these men with their overbearing self-confidence before the war is as impressive as the sight of the obliterated villages of Pozières and Contalmaison, and of the ruined heaps of Lens. These men seem to have lost their nationality, are said to be largely sending their capital abroad, and their very hopelessness and " unresisting apathy is a distinct danger, as it removes factors tending to stability." The report further states that this description does not apply universally, for many of those who served with the army and were consequently well fed will survive and come to the top in the case of a general breakdown of the civilisation of Germany. One further deduction, it seems to us, Prof. Starling might have made, and this applies to the producers, which number about one-third of the population and include practically the whole class of large landowners or Junkers who have been largely accused of starting the war—viz., that they have been well fed the whole time, and are therefore in a position, as soon as they can become organised, to play the same leading rôle as before the war, and whether for good or evil remains to be seen.

### The Physical Effects.

Turning to the physical effects of the underfeeding, they manifest them broadly in three ways—(1) increase of deaths, (2) decrease of births, and (3) increase of disease. The general death-rate increased from 14.8 per 1,000 of the population in 1914 to 23.5 in 1918. The official statistics show an increased number of about 760,000 deaths among the civil population for the four years 1914-18 over the number to be expected from pre-war rates. The birth-rate decreased from 27 per 1,000 in 1914 to 14.5 in 1918, so if you put the loss through the increased deaths (2.0 millions) and decreased births (3.5 millions) together, it means a net loss of 5.5 millions. The increase of disease was specially marked in the case of tuberculosis and rickets. The former affected largely the adult population, and the latter the children. In Prussia tubercle of the lungs increased two and a-half times, and is now equal to what it was forty years ago, and in Kattowitz the death-rate from tubercle increased six times. In children the death-rate from two to six years increased from 1913 to 1917 49.3 per cent., and this was largely due to the almost complete withdrawal of milk. Before the war Berlin received 1,250,000,000 litres of milk per day, during it only 225,000 litres. As a result of this and the small amount of butter available, rickets became prevalent in practically all classes, resistance to infection was greatly diminished, and numerous cripples may be expected as the result of bone deformities caused during the time of shortage.

### A Diminished Birth-rate.

It is pointed out that one contributory cause of the diminished birth-rate, besides the absence of men at the Front, was the underfeeding of parents, and the report adds in this connection a most significant sentence: "*In spite of the condition of malnutrition of the mothers, the children, when born, are normal,*" though it is added their further development is seriously interfered with by lack of nourishment of the mother and the shortage of milk. These facts may help to put some restraint on those who would spend much money on ante-natal work to the detriment of natal and post-natal work, for " Nature," when she

The previous article appeared on September 27, p. 635.

**Food Conditions in Germany**—(*continued*).

has sole charge of the offspring, as she has before birth, seems to do her work thoroughly and bring forth a healthy child; it is later, when the child comes under "nurture," that the purely human element seems to fail.

Another lesson is conveyed in the next paragraph, and that is that though the children were often pale and emaciated when sent to school, and inattentive through lack of food, even in "the lower-class schools they were *clean and neatly dressed*."

The last part of the report deals with the immediate future, and shows that no amelioration of the food difficulties of Germany is to be expected in the next year unless the Allies come to the rescue, and "that Germany stands face to face with a catastrophe which may involve the death of millions by famine and disease unless she can obtain by importation sufficient food to make up her deficit." Then follow elaborate calculations of the amount required—the special needs at this time being fat and animal protein. It is to be hoped, therefore, that the Allies will come to the rescue of our fallen foe.

